# Influence of Progesterone-Treatment Length and eCG Administration on Appearance of Estrous Behavior, Ovulatory Success and Fertility in Sheep

**DOI:** 10.3390/ani9010009

**Published:** 2018-12-26

**Authors:** Paula Martinez-Ros, Alejandro Rios-Abellan, Antonio Gonzalez-Bulnes

**Affiliations:** 1 Departamento de Produccion y Sanidad Animal, Facultad de Veterinaria, Universidad Cardenal Herrera-CEU, CEU Universities, C/ Tirant lo Blanc, 7, Alfara del Patriarca, 46115 Valencia, Spain; rioabeale@alumnos.uchceu.es; 2 Departamento de Reproduccion Animal, Instituto Nacional de Investigación y Tecnología Agraria y Alimentaria (INIA), Avda. Puerta de Hierro s/n. 28040 Madrid, Spain; bulnes@inia.es; 3 Departamento de Toxicologia y Farmacologia, Facultad de Veterinaria, Universidad Complutense de Madrid, Ciudad Universitaria s/n. 28040 Madrid, Spain

**Keywords:** CIDR, eCG, estrus synchronization, Fertility, Ovulation, Sheep

## Abstract

**Simple Summary:**

This study examines the pre-ovulatory and ovulatory events (in terms of the timing of the onset of estrous and subsequent ovulation) and the yields obtained (in terms of ovulation rate, progesterone secretion, and fertility) after insertion of Controlled Internal Drug Release (CIDR) for five, six, seven, or fourteen days, with or without equine chorionic gonadotrophin (eCG).

**Abstract:**

The present study characterizes, for sheep, the occurrence and timing of the onset of estrus behavior and ovulation and the yields obtained (ovulation rate, progesterone secretion, and fertility) after Controlled Internal Drug Release (CIDR) insertion for five, six, seven, or fourteen days, with or without equine chorionic gonadotrophin (eCG) in ewes of the Segureña meat breed. All the treatments showed above 80% of females displaying estrus, but the onset of estrus was earlier and more synchronized when using eCG and, among groups with eCG, onset of estrus was earlier in the sheep treated for 14 days than in the short-term treatments (*p* < 0.05 for all). Administration of eCG after either short- or long-term treatments assured the occurrence of fertile ovulations in all the animals. Conversely, ovulatory success without eCG was found to be dependent on treatment length, with a high percentage of animals ovulating after five days of treatment (83.3%) and very low percentages after treatment for six or seven days (40% and 20%, respectively). Ovulation rate and progesterone secretion were similar among animals ovulating, but ovulation failures predetermined the fertility yields obtained in response to the treatments. Hence, the best results were found after treatment for 14 days plus eCG, and for 5 days without eCG (83.3 for both, *p* < 0.05 when compared to the other groups with different treatment lengths and with or without eCG).

## 1. Introduction

Synchronization of estrus and ovulation for fixed-time artificial insemination (FTAI) in sheep is mostly based on the insertion of intravaginal devices containing either progesterone CIDR (Controlled Internal Drug Release) inserts or progestagens (sponges impregnated with fluorogestone acetate (FGA) or medroxyprogesterone acetate (MAP)). At device removal, a single intramuscular injection of equine chorionic gonadotrophin (eCG) is added for inducing ovulation and synchronizing its time of appearance among animals in the same lot [1].

Administration of eCG is, therefore, essential for FTAI. However, there is currently a highly active movement in European countries against the use of eCG. The pressure group has forced the production of eCG to move out of the European Union and aims to get its use banned in animal production. The European Union (EU) government’s opinion is in favor of continuing eCG use, but the social opinion against companies trading with eCG may force them to discontinue the imports. Hence, it is necessary to look for alternative protocols for the induction and synchronization of estrus and ovulation which would not include eCG. A first step would be the generation of healthy growing preovulatory follicles able to induce estrus and able to reach a fertile ovulation after withdrawal of the progestative treatment; for FTAI purposes, such events might be highly synchronous among treated animals.

Classically, from its development in the 1960s [2], the progestative treatment is maintained for 12–14 days to surpass the half-life of a possible corpus luteum present at the ovaries at the time of device insertion. The fertility obtained after applying such a protocol was quickly reported to be lower than after natural estrus, and successive studies evidenced alterations in the patterns of luteinizing hormone (LH) release [3,4] and the quality of the subsequent ovulations [5]. These alterations were later related to the prolonged time of treatment permanence, and these findings supported the use of short-term (5–7 days) progestative treatments [6,7,8]. Currently, short-term protocols are recognized to be equally as effective as long-term protocols for inducing estrus, ovulation, and fully functional corpora lutea [9], with lower incidence of vaginal infections [10] and higher fertility yields [11].

The advent of ovarian ultrasonography in the 1990s [12] allowed for the characterization of the follicular turnover during the estrous cycle [13] and also gave evidence of the success of the short-term treatments. In studies using CIDR [14], the screening of the effects of exogenous progesterone on follicular growth showed that the high blood progesterone concentrations obtained at device insertion lowered the secretion of LH. Such a decrease in LH availability after CIDR insertion, being the hormone which supports dominant follicles [15], causes atresia of large follicles present in the ovaries and promotes follicular turnover, leading to the appearance of new preovulatory follicles that reach their maximum diameter after 5–7 days of CIDR insertion. Hence, removal of the progestative treatment at such a period would assure the presence of a healthy growing preovulatory follicle able to induce estrus and reach fertile ovulation [11].

In view of these considerations, the objective of the present study was to characterize the pre- ovulatory and ovulatory events (in terms of the timing of the onset of estrous and subsequent ovulation) and the yields obtained (in terms of ovulation rate, progesterone secretion, and fertility) after insertion of CIDR for five, six, seven, or fourteen days, with or without eCG. These results have direct implications for evaluating effectiveness of the proposed practice of using short-term CIDR protocols without eCG.

## 2. Material and Methods

### 2.1. Animals and Experimental Design

The trial was carried out during the end of breeding season (April) and involved a total of 58 multiparous meat ewes (of the Segureña breed, 2–5 years old). All the sheep were cycling as checked by ovarian ultrasonography just prior to starting the experiment. Sheep were maintained outdoors with access to indoor facilities at the experimental farm of the Cardenal Herrera University in Naquera (Valencia, Spain; latitude 39°N), which met local, national, and European requirements. The experiment was performed according to the Spanish Policy for Animal Protection RD53/2013, which met the European Union Directive 2010/63/UE about the protection of animals used for research, and was specifically assessed and approved by the CEU Cardenal Herrera Committee of Ethics in Animal Research (report CEEA17/019).

Ovarian cyclic activity and ovulation were synchronized in all the animals by the insertion of one intravaginal, progesterone-loaded CIDR (CIDR^®^ Ovis, Zoetis, Madrid, Spain). Sheep were divided into eight groups according to the duration of the progesterone treatment (5, 6, 7, and 14 days) and the administration of a single intramuscular dose of 400 International Units of eCG (groups eCG; Foligon^®^, MSD Animal Health, Madrid, Spain) or the equivalent amount of physiological saline solution (groups SS) at CIDR removal. Hence, the groups were C5eCG (*n* = 6), C5SS (*n* = 6), C6eCG (*n* = 6), C6SS (*n* = 6), C7eCG (*n* = 6), C7SS (*n* = 6), C14eCG (*n* = 12), and C14SS (*n* = 10). To avoid the possible permanence of an active corpus luteum after the short-term treatments (5, 6, and 7 days), these groups were treated with an intramuscular injection of 5 mg of prostaglandin F_2α_ (Dinolytic^®^, Zoetis, Madrid, Spain) at CIDR withdrawal.

The variables evaluated during the induced follicular phase and the subsequent luteal phase were the percentage and timing of the onset of estrous behavior and ovulation, the number and functionality (in terms of progesterone secretion) of the induced corpora lutea, and the fertility rate.

### 2.2. Occurrence and Timing of Estrus Onset

Signs of estrous behavior were determined every 4 h from 12 to 60 h after CIDR withdrawal by the use of trained rams in the proportion of one ram/one ewe. The interval from treatment to the onset of the estrus was defined by the time elapsed between device removal and the first accepted mating.

### 2.3. Occurrence and Timing of Ovulation

Assessment of ovulation was performed every 4 h from 48 to 80 h after CIDR withdrawal. In each scanning, the ovaries of the sheep were examined by transrectal ultrasonography using a real-time, B-mode scanner (Aloka SSD 500, Aloka Co. Ltd., Tokyo, Japan) fitted to a 7.5 MHz linear-array probe, as previously described and validated in our laboratory [16]. Similarly to a previous study, the disappearance of large anechoic structures (i.e., ovulatory follicles) recorded in a previous ultrasonography was considered to be presumptive ovulation [17].

### 2.4. Ovulation Rate and Corpora Lutea Functionality

In all the animals, the number of corpora lutea was determined by ultrasonography at Day 12 of the induced estrous cycle. The luteal functionality was evaluated in terms of progesterone secretion by drawing blood samples coincidentally with ultrasound scanning and processing them as described above. Plasma progesterone concentrations were measured using a commercially available, direct solid-phase radioimmunoassay kit (PROG-CTRIA, IBA Molecular, Madrid, Spain). Sensitivity of the assay was 0.05 ng/mL, and inter- and intra-assay variation coefficients were 4.5 % and 3.5 %, respectively.

### 2.5. Fertility

The fertility rate was assessed by transrectal ultrasonography at Day 45 after CIDR withdrawal, using a real-time scanner (Aloka SSD 500, Aloka Co. Ltd., Tokyo, Japan) fitted to a 7.5MHz linear-array probe.

### 2.6. Statistical Analysis

Statistical analysis was performed using SPSS^®^ 22.0 (IBM Corporation, New York, NY, USA). The effects of the treatment length (five, six, seven, or fourteen days) and eCG injection (yes or no) on the occurrence and onset of estrous behavior and ovulation, ovulation rate, progesterone secretion, and fertility were assessed by analyses of variance (ANOVA). Statistical analysis of results expressed as percentages was performed after arcsine transformation of the values for each individual percentage. All results are expressed as mean ± SEM and the statistical significance was accepted at *p* < 0.05.

## 3. Results

### 3.1. Occurrence and Timing of Estrous Behavior

All the treatments showed a high percentage, above 80%, of females displaying estrous behavior in response to the treatment (Table 1), without any significant effect from eCG injection or treatment length.

However, there were significant effects from both eCG injection and treatment length on the timing of onset of the estrus. Overall, the administration of eCG induced an earlier and more synchronized onset of estrus, the differences being statistically significant when comparing the groups C7eCG vs. C7SS (*p* < 0.05) and C14eCG vs. C14SS (*p* < 0.05). Treatment length affected the onset of estrus only in the groups treated with eCG, inducing an earlier onset in the group C14eCG than in the short-length treatments (*p* < 0.05 for all). However, no significant differences were found when comparing treatments without eCG.

### 3.2. Occurrence and Timing of Ovulation

The eCG injection had a significant effect on the occurrence of ovulation (Table 1). All the sheep treated with eCG and showing estrous behavior ovulated after the CIDR treatment. Conversely, not all the animals in the groups treated with saline solution ovulated; the percentage of animals ovulating was highest in the group C5SS (83.3%) and very low in the groups C6SS (40%, *p* < 0.01) and specially C7SS (20%, *p* < 0.005). Ovulations were not detected in the animals which did not show estrous behavior.

The low number of sheep ovulating in the groups C6SS and C7SS did not allow for statistical comparisons. In the remaining groups, the administration of eCG induced an earlier ovulation in the group C14eCG than in C14SS (around 10 h earlier, *p* < 0.05), whilst conversely, there were no differences between the groups C5eCG and C5SS (*p* = 0.854)

Treatment length in the groups receiving eCG administration affected the timing of ovulation, which was around 15 h earlier in the group C14eCG than in all the short treatments (*p* < 0.005 for all); there were no differences among the three short treatments.

### 3.3. Ovulation Rate and Corpora Lutea Functionality

There were no significant effects of either treatment length or eCG treatment on both the number of corpora lutea and the progesterone concentration in response to the treatment (Table 1).

### 3.4. Fertility

Fertility of ewes responding with ovulations to the progestative treatment was higher than 60% in all the groups, the percentage being higher in the short-term treatments without eCG. There were no significant differences among the groups, which may be due to the low number of animals in each group. Assessment of the percentage of pregnant ewes in relation to treated ewes showed significant differences among the groups (Table 1), with very low results in the groups treated with CIDRs for six and seven days without eCG (C6SS and C7SS, *p* < 0.05 when compared to the other groups) and better results in the groups treated for fourteen days plus eCG and for five days without eCG (C14eCG and C5SS, *p* < 0.05 when compared to the other groups).

## 4. Discussion

The results of the present study indicate that the administration of CIDR-based protocols, independently of the addition of eCG and the duration of the treatment, provokes the appearance of estrous behavior after device withdrawal in most of the treated sheep. However, both the eCG injection and the treatment length have significant effects on the timing and grouping of estrus and subsequent ovulation.

In brief, eCG administration advanced and grouped the appearance of estrous behavior and induced ovulation in all the ewes that exhibited estrus. In the sheep treated with eCG, onset of estrus was earlier in the long-term treatment than in any of the short-term treatments; such a difference was not found in the sheep without eCG. Assessment of ovulation showed that, conversely to groups with eCG, groups without eCG had a high incidence of ovulatory failures; such an incidence was determined by the length of the CIDR treatment, being higher in groups treated for six and seven days than in the other groups.

Afterwards, all the sheep ovulating had corpora lutea of adequate morphological quality and progesterone secretion. The information about fertility yields obtained in the current work must only be considered informative and very preliminary, due to the low number of animals in each group. The design of the present study was mainly focused on the effects of treatment length and eCG addition on the characteristics of estrus and ovulation rather than on fertility, and the experimental designs for studying these variables are not quite compatible. In fact, evaluation of changes in fertility requires a large number of animals, whilst evaluation of timing of estrus and ovulation requires a high number of successive samples which are obtained from a small number of animals. However, in the present study, the fertility obtained may account for supporting the previous results on ovulatory success, highlighting the poorer yields in the groups treated for six and seven days without eCG. On the other hand, the group treated for five days without eCG showed similar ovulatory efficiency and fertility, as did the group treated with the classical protocol, including 14 days of CIDR insertion and eCG. In fact, a recent unpublished study developed on 150 ewes under field conditions showed similar levels of fertility between sheep treated with CIDR for 14 days plus eCG, and for five days without eCG (51 and 48%, respectively); in that trial, fertility was higher when using CIDR for five days and eCG (69%) than in the previously cited groups.

In summary, the results of the present study support previous knowledge on the beneficial role of eCG treatment on ovulatory success and subsequent fertility, and emphasize the negative consequences of eCG unavailability for sheep productivity. The present trial was carried out during the breeding season, so even worse results may be expected for treatments during seasonal anestrous, when gonadotrophin secretion and ovulation processes are depressed [18].

Hence, for the prevention of the possible unavailability of eCG, progesterone-based protocols need to be refined and improved for safeguarding enough good outcomes. Our results indicate that a treatment based on five days of exposure to progesterone may constitute a reliable alternative, which obviously needs to be confirmed by further studies and in-field validation. We can hypothesize that the higher efficiency of the 5-day treatment when compared to 6-, 7-, or 14-day protocols may be related to the patterns of follicular growth described in previous studies.

The CIDR provides high blood progesterone concentrations immediately after its insertion, due to its efficient release kinetics [19]. High plasma progesterone concentrations reduce the secretion of LH at the pituitary [20] and the maintenance of large follicles at the ovary is highly dependent on LH support [15]; hence, such large follicles become atretic in case of shortage of the hormone, giving way to the recruitment of a new follicular wave. Early studies indicated that high levels of progestagens affect viability of large follicles and increase follicular turnover [21,22]. In fact, these effects are found, not only after maintained exposure to progesterone/progestagens for several days, but also after the administration of a single progesterone injection [23] which induces high plasma progesterone concentrations for only 20–24 h [24].

From the very first studies on sheep folliculogenesis, it has been known that when the largest follicles present in the ovaries decrease in size at the end of a follicular wave, the following preovulatory follicle emerges from the pool of smaller antral follicles [25,26]. Such a follicle is in its active growing phase for the following four days [27,28] to reach its maximum diameter at the fifth day [29]. If we extrapolate this to the end of a follicular wave caused by the insertion of a CIDR, we would have a new growing preovulatory-size follicle from five days after device insertion, as reported by Menchaca and Rubianes [14]. Our current results suggest that this preovulatory-size follicle would be in its active growing phase five days after CIDR insertion. However, it would be entering into the static phase at six or seven days after CIDR insertion and, therefore, its ability to ovulate without eCG stimulation would be compromised. Induction of a follicular phase by device withdrawal in animals in which the largest follicles are in the static or early-atretic phase may cause ovulatory failure due to deficiencies in their quality and stereidogenic ability [30]. At first glance, the difference of a couple of days in the duration of progestative treatment may seem to be insignificant. However, we need to bear in mind that differences in size between dominant and subordinate follicles are very small (2–3 mm) and periods of effective dominance are very short (2–3 days) in sheep [31].

These patterns of follicular dynamics after inducing high plasma progesterone levels would also explain the delay of estrus and ovulation in short-term treatments when compared to long-term treatments, due to the need for the development of a new follicular wave from which ovulatory follicles arise. The same effect has been found after the administration of progestagen-impregnated sponges [9] and even after the administration of a single intramuscular dose of progesterone [23]. Such an effect is not critical when applying natural mating, but needs to be taken into account when implementing artificial insemination at a fixed time.

## 5. Conclusions

Administration of eCG after either short- or long-term CIDR treatments assures the occurrence of fertile ovulations, whilst ovulatory success after protocols without eCG is dependent on the duration of the CIDR treatment; the best results being the yield after five days of device insertion.

## Figures and Tables

**Table 1:** Percentage and timing of the occurrence of estrus and ovulation, number of corpora lutea, progesterone secretion, and fertility in ewes treated with Controlled Internal Drug Release (CIDR) for five, six, seven, and fourteen days (groups C5, C6, C7, and C14, respectively), with equine chorionic gonadotrophin or physiological saline solution (groups eCG and SS, respectively).

	C5eCG (*n* = 6)	C5SS (*n* = 6)	C6eCG (*n* = 6)	C6SS (*n* = 6)	C7eCG (*n* = 6)	C7SS (*n* = 6)	C14eCG (*n* = 12)	C14SS (*n* = 10)
Occurrence of estrus (%)	100	100	100	83.3	83.3	83.3	91.7	100
Time of onset of estrus after CIDR removal (h)	40.8 ± 8.0 ^1^	45.0 ± 5.7	42.5 ± 5.9 ^1^	52.4 ± 14.5	41.6 ± 6.7 ^a,1^	58.2 ± 8.9 ^b^	30.9 ± 9.5 ^a,2^	45.3 ± 16.3 ^b^
Occurrence of ovulation (%)	100	83.3 ^1^	100	40 ^2^	100	20 ^3^	100	70 ^1^
Time of ovulation after CIDR removal (h)	71.8 ± 7.3 ^1^	72.6 ± 5.8	73.8 ± 5.9 ^1^	69.0 ± 7.1	70.6 ± 6.5 ^1^	77	56.7 ± 6.9 ^a,3^	66.7 ± 8.3 ^b^
Number of corpora lutea	1.7 ± 0.5	1.4 ± 0.5	2.2 ± 0.8	2.0 ± 1.4	1.8 ± 0.4	2	2.2 ± 0.9	1.9 ± 0.7
Plasma progesterone concentration (ng/ml)	5.6 ± 2.9	4.5 ± 3.3	5.0 ± 3.1	3.3 ± 1.0	3.8 ± 1.6	5.8	5.7 ± 2.2	3.9 ± 1.5
Fertility rate with regard to ewes ovulating (%)	66.7	100	80	100	60	100	90.9	85.7
Fertility rate with regard to treated ewes (%)	66.7	83.3	80	33.3	50	16.7	83.3	60

Different superscripts indicate significant differences among treatments with different length (numbers; 1≠2: *p* < 0.01; 1≠3: *p* < 0.005) and with or without eCG (letters; a≠b: *p* < 0.05).
